# A Amazônia desenvolvimentista em foco: dimensões

**DOI:** 10.1590/S0104-59702026000100020

**Published:** 2026-06-29

**Authors:** Iane Maria da Silva Batista

**Affiliations:** i Professora, Programa de Pós-graduação em Ciência da Informação/Universidade Federal do Pará. iane@ufpa.br


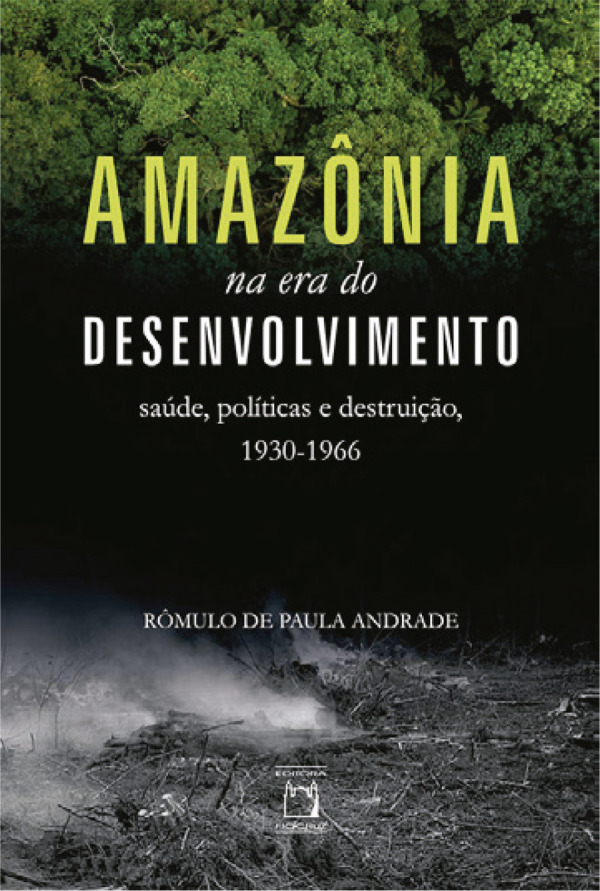


O livro *Amazônia na era do desenvolvimento: saúde, políticas e destruição (1930-1966)*, do historiador Rômulo de Paula Andrade (2024), é resultado de uma trajetória de pesquisa dedicada à história das relações entre saúde e meio ambiente no contexto de projetos desenvolvimentistas elaborados e executados no Brasil República. O tema é abordado em sete capítulos nos quais o leitor é apresentado a múltiplos personagens, programas e instituições que pensaram, planejaram e intervieram sobre o espaço amazônico entre 1930 e 1966. A obra descortina e interpreta interesses, confluências e divergências políticas, ideológicas, econômicas e científicas atuantes em uma arena discursiva permeada pela reedição de mitos e propagação de estereótipos sobre a Amazônia, como insalubridade do clima, vazio demográfico, monotonia alimentar e/ou a acomodação das populações regionais a um meio físico neutralizador de uma economia “produtiva”.

A pesquisa reflete o diálogo com uma historiografia consistente e atualizada e a consulta a um amplo repertório de fontes documentais em diferentes fundos arquivísticos no Brasil e nos Estados Unidos envolvendo depoimentos orais, correspondências, periódicos, relatórios, inquéritos administrativos e legislações, além de romances e cinedocumentários. A análise revela uma história coerente, dolorosa e entrelaçada com acontecimentos do presente, por exemplo, quando o autor relaciona o assassinato do indigenista Bruno Araújo e do jornalista britânico Dom Phillips em 2022 a um projeto de Amazônia idealizado e executado pelos diversos agentes apresentados no texto e que, com algumas (re)atualizações, persiste.

Os anos 1930-1945 são apresentados pelas lentes do aparato cultural e ideológico de Getúlio Vargas instrumentalizado pelo Departamento de Imprensa e Propaganda, especialmente nas páginas da revista *Cultura Política*. Aspectos simbólicos da Marcha para o Oeste e do Discurso do Rio Amazonas são interpretados como parte importante de um processo de reescrita da história regional do país. A análise do pensamento e da atuação de profissionais de saúde pública, como Evandro Chagas e João de Barros Barreto, revela conexões internacionais, negociações, acordos e tensões envolvendo a criação de instituições de saúde e saneamento na Amazônia. As posições médicas, científicas e políticas explicitadas pelo autor nos capítulos iniciais lançam novas perspectivas sobre eventos emblemáticos na história brasileira da primeira metade do século XX, como a Batalha da Borracha.

A Amazônia de meados dos anos 1940 e da década de 1950 – em especial, a criação, a atuação e o desmonte de sua agência desenvolvimentista pioneira, a Superintendência de Valorização Econômica da Amazônia – é interpretada a partir da sistematização de diversas perspectivas conceituais do desenvolvimento, palavra-chave para se entender as opções feitas pelo Estado brasileiro naquele contexto histórico de Guerra Fria, bem como os desdobramentos socioambientais que elas produziram nas décadas seguintes. Ao discutir a atuação de agências internacionais na região como a Organização das Nações Unidas para a Alimentação e a Agricultura e a formulação de programas de saúde visando transformar os padrões alimentares e sanitários locais, Andrade (2024) demonstra os descompassos entre as intenções de médicos, cientistas e políticos e a efetividade de medidas pontuais que se tentou implantar nas localidades amazônicas, com destaque para as iniciativas de erradicação da malária.

Ao interrogar os processos históricos envolvendo a concepção e construção da rodovia Belém-Brasília no limiar dos anos 1960, o livro aponta as perspectivas otimistas de progresso e integração nacional materializadas na estrada e alimentadas pelo “espírito bandeirante” do governo de Juscelino Kubitschek (1956-1961). A interpretação das visões de natureza e do papel da Amazônia no desenvolvimentismo brasileiro, que emergem das fontes produzidas sobre o empreendimento rodoviário naquele contexto, é uma contribuição relevante do autor à historiografia sobre esse período.

Em consonância com a historiografia contemporânea dedicada ao tema do desenvolvimentismo, Andrade (2024) aponta as contradições dos projetos pensados para a Amazônia, especialmente a exclusão das populações locais dos processos decisórios que afetaram suas vidas de muitas maneiras, quase sempre sob o signo da violência, ainda que casos pontuais tenham exigido uma adaptação de discursos e práticas estatais às possibilidades efetivas de ação. A contextualização histórica delineada no livro mostra como as tentativas de “conhecer” a região para nela poder intervir não dimensionavam a complexidade da sociobiodiversidade amazônica.

A esse respeito, é significativa a ausência, nas fontes pesquisadas sobre os aspectos alimentares e nutricionais da região, de referências ao açaí (*Euterpe oleracea* Mart.), considerando que o consumo regular dos frutos dessa palmeira por populações amazônicas estuarinas tem considerável trajetória. De acordo com Leila Mourão (2011), pesquisas acerca da composição bioquímica do açaí vêm sendo realizadas desde 1930, identificando nele elevado teor nutricional formado por proteínas, açúcares, fibras, ferro, potássio, cálcio, vitamina B e antocianinas. Uma investigação sobre essa lacuna nos escritos dos homens de ciência e de Estado que se preocuparam com as questões nutricionais da Amazônia no período estudado pelo autor pode vir a ser um desdobramento interessante de pesquisa.

A historiografia do planejamento do desenvolvimento regional amazônico, mais centrada no contexto pós-Operação Amazônia, lançada pelo então presidente Castello Branco em dezembro de 1966, ganha uma importante contribuição com *Amazônia na era do desenvolvimento: saúde, políticas e destruição (1930-1966)*. Os estudiosos dos grandes projetos agropecuários, mineradores, hidrelétricos e colonizadores executados pelos governos militares na região podem ampliar suas perspectivas teóricas e metodológicas sobre o tema a partir das fontes documentais analisadas pelo autor e de suas consistentes interpretações a respeito do processo histórico regional, nacional e internacional que “preparou o terreno” para a viabilização daqueles empreendimentos.

A obra também representa importante fonte de conhecimento ao leitor não especialista, interessado em compreender a história da Amazônia dos séculos XX e XXI, suas continuidades e descontinuidades. No contexto dos preparativos para a trigésima Conferência das Partes (COP-30), realizada em Belém do Pará, em novembro de 2025, em pleno “coração” da Amazônia brasileira, a imprensa repercutiu diversas manifestações públicas de comunidades periféricas afetadas pelos dejetos de obras de infraestrutura em áreas nobres da cidade (Vazquez, 7 abr. 2025). A população belenense também assistiu, perplexa, à instalação de árvores de plástico fixadas em estrutura de vergalhões metálicos ao longo de algumas das vias urbanas que estavam sendo preparadas para o evento, iniciativa supostamente inspirada em um projeto urbanístico de Cingapura (Bessa, 1 abr. 2025). A desfaçatez desses episódios é reveladora da persistência de um padrão estatal que não pensa a Amazônia a partir de suas próprias singularidades. Conhecer e compreender, pela hábil narrativa de Rômulo de Paula Andrade, os discursos e as práticas de médicos, políticos, cientistas, intelectuais, jornalistas que pensaram e atuaram sobre o território amazônico entre 1930 e 1966, pode ajudar a desbravar os complexos e polissêmicos caminhos da Amazônia contemporânea.
